# Serum Metabolomic Characterization of Liver Fibrosis in Rats and Anti-Fibrotic Effects of Yin-Chen-Hao-Tang

**DOI:** 10.3390/molecules21010126

**Published:** 2016-01-21

**Authors:** Hongyang Zhang, Xiaoning Wang, Ping Hu, Wenjun Zhou, Min Zhang, Jia Liu, Yuerong Wang, Ping Liu, Guoan Luo

**Affiliations:** 1 School of Chemistry and Molecular Engineering, East China University of Science and Technology, Shanghai 200237, China; hongyang_zhang@ecust.edu.cn (H.Z.); wangyuerong@ecust.edu.cn (Y.W.); 2 E-Institute of Shanghai Municipal Education Committee, Shanghai University of Traditional Chinese Medicine, Shanghai 201203, China; wxntcm@126.com (X.W.); wjzhou678@163.com (W.Z.); fionisly@hotmail.com (J.L.); 3 School of Pharmacy, East China University of Science and Technology, Shanghai 200237, China; zhangm@ecust.edu.cn (M.Z.); luoga@mail.tsinghua.edu.cn (G.L.); 4 Key Laboratory of Liver and Kidney Diseases (Ministry of Education), Institute of Liver Diseases, Shuguang Hospital, Shanghai 201203, China; 5 Department of Chemistry, Tsinghua University, Beijing 100084, China

**Keywords:** serum metabolomics, liver fibrosis, Yin-Chen-Hao-Tang, anti-fibrotic effects, oxidative stress, lipid peroxidation, ultra-performance liquid chromatography-time-of-flight mass spectrometry, partial least squares-discriminant analysis

## Abstract

Yin-Chen-Hao-Tang (YCHT) is a famous Chinese medicine formula which has long been used in clinical practice for treating various liver diseases, such as liver fibrosis. However, to date, the mechanism for its anti-fibrotic effects remains unclear. In this paper, an ultra-performance liquid chromatography-time-of-flight mass spectrometry (UPLC-TOF-MS)-based metabolomic study was performed to characterize dimethylnitrosamine (DMN)-induced liver fibrosis in rats and evaluate the therapeutic effects of YCHT. Partial least squares-discriminant analysis (PLS-DA) showed that the model group was well separated from the control group, whereas the YCHT-treated group exhibited a tendency to restore to the controls. Seven significantly changed fibrosis-related metabolites, including unsaturated fatty acids and lysophosphatidylcholines (Lyso-PCs), were identified. Moreover, statistical analysis demonstrated that YCHT treatment could reverse the levels of most metabolites close to the normal levels. These results, along with histological and biochemical examinations, indicate that YCHT has anti-fibrotic effects, which may be due to the suppression of oxidative stress and resulting lipid peroxidation involved in hepatic fibrogenesis. This study offers new opportunities to improve our understanding of liver fibrosis and the anti-fibrotic mechanisms of YCHT.

## 1. Introduction

Liver fibrosis is a prevalent chronic liver disease characterized by the increased hepatic deposition of extracellular matrix proteins [1]. Liver fibrosis itself causes no symptoms, but the advanced fibrosis can lead to irreversible cirrhosis or liver cancer, and finally often requires liver transplantation [2]. Previous reports have indicated that liver fibrosis is potentially reversible, so the early diagnosis and intervention of this disease have important clinical implications [3]. Until now, numerous works have centered on gene expression, protein function, as well as conventional pathophysiological studies on the liver fibrosis [4,5]. The mechanisms for liver fibrosis have been dramatically elucidated but are still not fully understood. As such a complex and multifactorial process, liver fibrosis involves not only specifically expressed genes and proteins, but also the changes of endogenous metabolites, and to investigate the metabolic variations might be expected to provide new insights into the pathogenesis of fibrotic process [6].

Since liver fibrosis is shown to be reversible, efforts should be made to search for effective therapeutic approaches. Unfortunately, no established anti-fibrotic therapies are currently available [7]. Traditional Chinese medicine (TCM), owing to its good efficacy and few side effects, has displayed unique features in the treatment of liver diseases for thousands of years. Varieties of herbal extracts and formulae have been shown to exhibit anti-fibrotic properties [8,9]. For example, Yin-Chen-Hao-Tang (YCHT) is a classic Chinese herbal formula that consists of three medicinal materials as follows: *Artemisia capillaries*, *Fructus gardenia*, and *Radix et Rhizoma Rhei* [10]. Our previous results have revealed that YCHT has protective effects against an experimental model of liver fibrosis through the inhibition of hepatic stellate cell (HSC) activation [11]. However, due to the complex interactions between the active components and biological systems, little is known about the precise mechanisms of the anti-fibrotic effects offered by YCHT. Systemic studies are therefore required to provide a comprehensive evaluation of the efficacy of YCHT.

Metabolomics, which aims to analyze the global metabolic perturbations in biological systems, has been increasingly used as an efficient approach for exploring the potential mechanisms of diseases and assessing the therapeutic effects of drugs [12,13]. A number of analytical tools have been applied in metabolomics, such as nuclear magnetic resonance (NMR), gas chromatography-mass spectrometry (GC-MS) and ultra-performance liquid chromatography-mass spectrometry (UPLC-MS) [14]. Among these techniques, the UPLC-MS has gained more applications due to its improved resolution and sensitivity for non-volatile metabolite analysis without requiring derivatization [15]. Our recent urinary metabolomic studies based on GC-MS reported two TCM formulae with anti-fibrotic effects by regulating the dysfunction of relevant metabolisms in rats [16,17]. However, the serum metabolic profiling of liver fibrosis remains poorly understood and no metabolomic assessment of the anti-fibrotic effects of YCHT has been published.

Here, we conducted a metabolomic study to characterize the dimethylnitrosamine (DMN)-induced liver fibrosis in rats and evaluate the therapeutic effects of YCHT. Serum samples collected from rats in control, model and YCHT-treated groups were analyzed by using ultra-performance liquid chromatography-time-of-flight mass spectrometry (UPLC-TOF-MS). Partial least squares-discriminant analysis (PLS-DA) was further utilized to identify the fibrosis-related metabolic alterations and investigate the anti-fibrotic mechanisms of YCHT.

## 2. Results and Discussion

### 2.1. Histological and Liver Function Examinations

**Figure 1:**
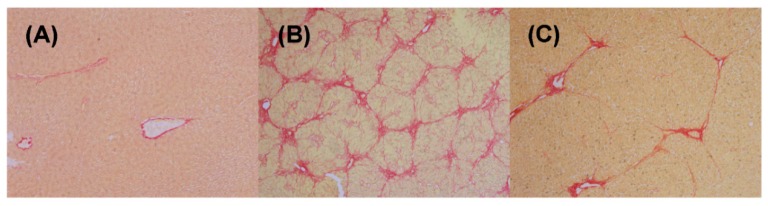
Representative histological photomicrographs of rat liver sections in the control (**A**); model (**B**) and YCHT group (**C**). Paraffin-embedded sections were stained with Sirius red (original magnification × 200).

In normal conditions, Sirius red staining was just observed in the centrilobular vein walls and portal area. Liver sections from the control group showed no fibrosis (Figure 1A). After DMN treatment, the livers of rats produced typical histological changes characterized by collagen deposition, architecture disruption and severe fibrous septa (Figure 1B). In addition, a fibrosis expansion of portal tracts with marked portal-to-portal and portal-to-central bridging and pseudolobuli formation was also observed [18]. However, the DMN-induced fibrotic rats with YCHT administration had significantly less histological collagen accumulation (Figure 1C). Liver fibrosis decreased markedly with reduced thickening of these collagen bundles in comparison with the model group.

The activities of serum enzymes, including ALT, AST, GGT and ALP, as well as the liver hydroxyproline content were markedly increased in the model rats as compared with the controls. Besides, the levels of Alb decreased and TBil significantly enhanced after DMN treatment. All these changes were obviously improved by YCHT intervention (Table 1). It could be concluded that the animal model of DMN-induced liver fibrosis in this study was successfully reproduced and YCHT treatment was demonstrated to have a positive role in ameliorating liver fibrosis.

**Table 1:** Serum biochemical parameters and liver hydroxyproline content of control, model, and YCHT-treated rats.

Parameter	Control	Model	YCHT
Serum			
ALT (U/L)	30.94 ± 8.07	90.07 ± 24.39 **	73.8 ± 9.89 ^∆∆^
AST (U/L)	55.06 ± 5.23	101.43 ± 16.53 **	83.3 ± 7.14 ^∆∆^
Alb (g/L)	30.48 ± 2.59	25.20 ± 2.42 **	27.65 ± 2.11 ^∆^
GGT (U/L)	95.22 ± 14.12	178.26 ± 37.11 **	127.39 ± 10.99 ^∆∆^
ALP (king/100 mL)	24.97 ± 4.03	92.76 ± 28.62 **	74.82 ± 13.75 ^∆^
TBil (mg/dL)	0.38 ± 0.11	0.58 ± 0.11 **	0.41 ± 0.06 ^∆∆^
Liver			
Hydroxyproline (μg/g)	210.48 ± 65.21	463.75 ± 156.72 **	307.91 ± 95.06 ^∆∆^

Data are represented as mean ± SD (*n* = 15 in each group). ** *p* < 0.01, *vs.* control group; ^∆^ *p* < 0.05, ^∆∆^ *p* < 0.01, *vs.* model group from one-way ANOVA analysis.

### 2.2. Metabolic Profiling and Multivariate Analysis

**Figure 2:**
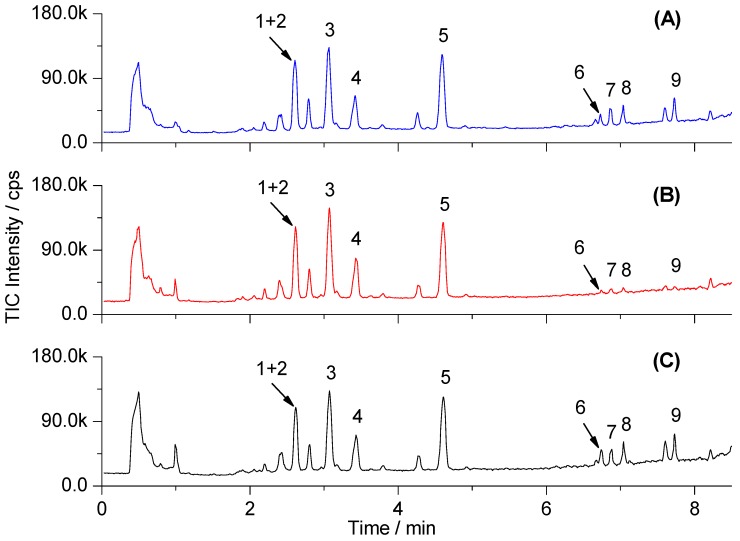
Representative UPLC-TOF-MS total ion chromatograms (TICs) of serum samples from control (**A**); model (**B**); and YCHT treated rats (**C**). Peak numbers of the identified metabolites are consistent with those in Table 2 and Table 3.

The UPLC, beneficial for the sub-2 μm column particles, can provide better resolution, higher sensitivity, and rapid separation as compared to the conventional HPLC. This system, combined with high-resolution TOF-MS, which enables accurate mass measurement, is suitable for global metabolomic analysis [19]. The UPLC-TOF-MS analysis in the ESI– mode was found to detect a greater number of serum metabolites than in the ESI+ mode. Therefore, the full scan detection was set as ESI− mode in our experiment. Under the optimized chromatographic and MS conditions, the typical total ion chromatograms (TICs) of serum samples in different groups are shown in Figure 2. The reproducibility data of the UPLC-TOF-MS method are listed in Table 2. The RSDs of intraday and interday variations for the representative compounds were lower than 8.5% and 10.5%, respectively, demonstrating that the method was reproducible for the metabolomic study.

**Figure 3:**
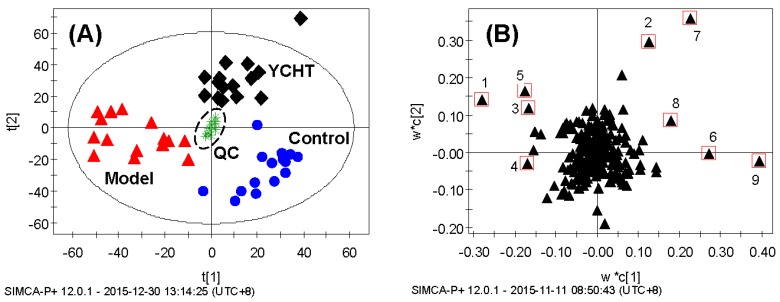
PLS-DA scores plot (**A**) and loadings plot (**B**) of rat serum data control group (blue dot, *n* = 15), model group (**red triangle**, *n* = 15), YCHT group (**dark diamond**, *n* = 15) and QC samples (**green star**, *n* = 10). The numbers of changed metabolites in loadings plot are consistent with those in Table 3.

To better visualize the metabolic variations among different groups, a supervised multivariate method—partial least squares-discriminant analysis (PLS-DA)—was used in this study. A well-fitted two-component PLS-DA model (*R*^2^*_X_* = 0.592, *R*^2^*_Y_* = 0.896, *Q*^2^ = 0.776) was then constructed. The tight location of QC samples in the scores plot (Figure 3A) demonstrated that the reliability of the analytical platforms was guaranteed. The scores plot showed a clear separation between the control and model groups in the first dimension, indicating a prominent fibrotic injury induced by DMN. Upon the treatment of YCHT, the serum metabolic profiles were found to move away from the models and exhibit a trend to recover to the controls, reflecting a potential protective effect of YCHT against liver fibrosis. In the corresponding loadings plot (Figure 3B), the distance of individual variables from the main cluster is positively related to their influence on the group separation, which means the compounds (variables) far away from the main cluster have greater impact on the classification. Additionally, the *VIP* (variable importance in the projection) value of each compound was calculated for selecting potential markers [20]. The compounds with lager *VIP* values represent higher contributions to the discrimination of different groups. As a result, nine metabolites with *VIP* values ≥ 2.00 were selected as candidate markers (Table 3).

**Table 2:** Reproducibility testing results of the UPLC-TOF-MS profiling method.

No.	*m/z* (amu)	Intraday Repeatability (*n* = 6)		Interday Repeatability (*n* = 9)	
		Mean Peak Area	RSD (%)	Mean Peak Area	RSD (%)
1	564.3295	2847	5.0	2834	6.7
2	588.3300	1914	5.7	1887	6.1
3	540.3293	5058	4.2	5055	5.2
4	566.3452	2664	6.6	2652	7.3
5	568.3609	4940	3.1	4937	5.2
6	327.2317	1230	7.4	1194	9.8
7	303.2333	1541	8.5	1483	10.5
8	279.2325	760	3.0	770	8.8
9	281.2478	1326	8.3	1310	9.7

Peak numbers of the selected compounds are consistent with those in Figure 2. Relative standard deviations (RSD %) were used to evaluate the method reproducibility.

**Table 3:** The identified candidate markers picked out by the PLS-DA loadings plot.

No.	*VIP* Value	*t*_R_ (min)	Selected Ion	*m/z* (amu)	Error (ppm)	Formula	Identification Results
1	2.86	2.54	[M + HCOO]^−^	564.3295	1.1	C_26_H_50_NO_7_P	Lyso-PC C18:2
2	2.41	2.58	[M + HCOO]^−^	588.3300	0.2	C_28_H_50_NO_7_P	Lyso-PC C20:4
3 *	2.39	2.98	[M + HCOO]^−^	540.3293	1.5	C_24_H_50_NO_7_P	Lyso-PC C16:0
4	2.00	3.43	[M + HCOO]^−^	566.3452	1.1	C_26_H_52_NO_7_P	Lyso-PC C18:1
5	2.41	4.53	[M + HCOO]^−^	568.3609	0.9	C_26_H_54_NO_7_P	Lyso-PC C18:0
6 *	2.48	6.73	[M − H]^−^	327.2317	2.1	C_22_H_32_O_2_	Docosahexaenoic acid
7 *	3.18	6.86	[M − H]^−^	303.2333	3.0	C_20_H_32_O_2_	Arachidonic acid
8 *	2.18	7.03	[M − H]^−^	279.2325	0.4	C_18_H_32_O_2_	Linoleic acid
9 *	3.36	7.72	[M − H]^−^	281.2478	1.1	C_18_H_34_O_2_	Oleic acid

Compounds with *VIP* values ≥ 2.00 were selected as candidate markers. * Compounds confirmed with authentic standards.

**Figure 4:**
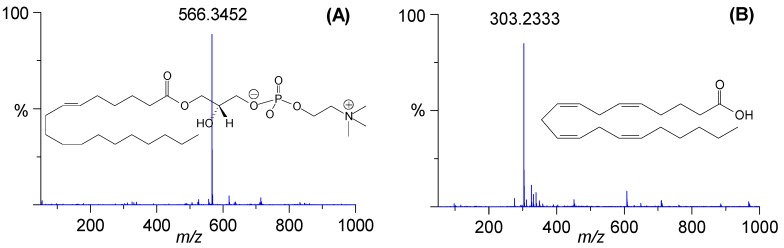
High-resolution TOF-MS mass spectra of the representative markers: (**A**) Lyso-PC C18:1 (No. 4) and (**B**) arachidonic acid (No. 7). The numbers of metabolites are consistent with those in Table 3.

**Figure 5:**
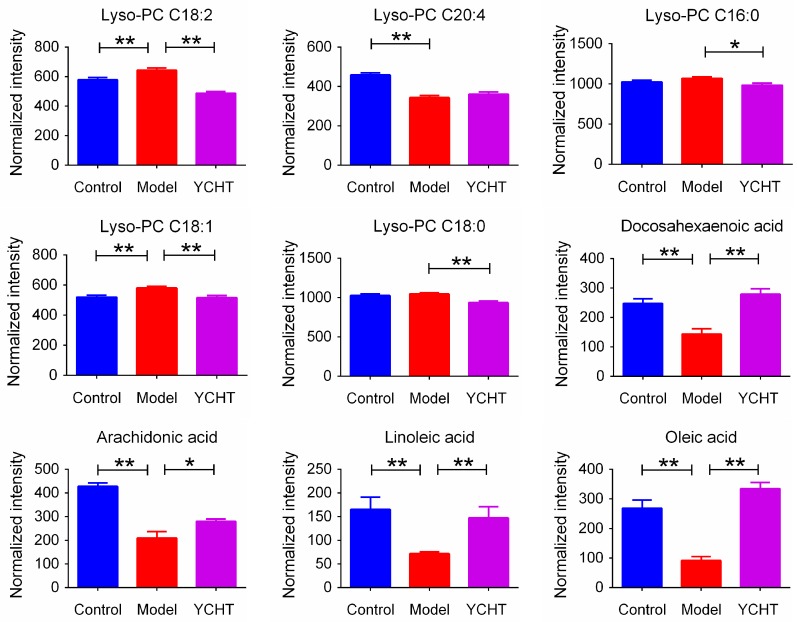
Altered levels of candidate markers in the control, model and YCHT treated rats. Data are represented as mean ± SEM (*n* = 15 in each group), with * *p* < 0.05 and ** *p* < 0.01 from one-way ANOVA analysis. Lyso-PC C16:0 and Lyso-PC C18:0 could not be considered as fibrosis-related markers since their levels in the control and model groups showed no statistical differences.

The TOF-MS provides both accurate mass (error within 5 ppm) and isotopic matching data, which are informative for biomarker identification. Based on our established method [21] and Human Metabolome Database (http://www.hmdb.ca/) searches, all candidate markers were identified including lysophosphatidylcholines (Lyso-PCs) and fatty acids (Table 3). Mass spectra of the representative marker metabolites are shown in Figure 4. Furthermore, these differential metabolites selected from the PLS-DA model were validated via one-way ANOVA analysis with a critical *p*-value of 0.05. Finally, seven metabolites were screened out as fibrosis-related markers and the two compounds of Lyso-PC C16:0 and Lyso-PC C18:0 were eliminated, since their levels in the control and model groups showed no statistical differences (Figure 5).

### 2.3. Fibrosis-Related Variations

In the identified markers, the serum levels of fatty acids were significantly lower in the fibrotic model rats than in the controls, while the levels of Lyso-PCs exhibited various changes between the two groups (Figure 5). These results suggest that an impaired lipid metabolism may play a crucial role in the pathogenesis of liver fibrosis.

**Figure 6:**
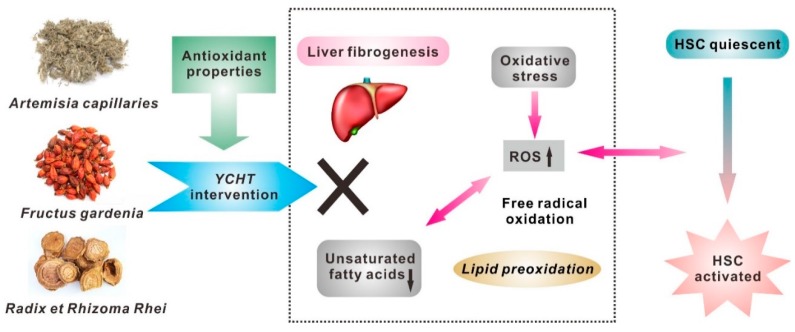
Proposed mechanistic pathways for the DMN-induced liver fibrosis and anti-fibrotic effects of YCHT. Upward arrowhead indicates up-regulation and downward arrowhead indicates down-regulation. HSC, hepatic stellate cell; ROS, reactive oxygen species; YCHT, Yin-Chen-Hao-Tang.

Liver fibrosis is a complex process which is mediated by the death of hepatocytes and the activation of HSCs [22]. HSCs are normally quiescent in the body, but the activated HSCs are responsible for the excess production of extracellular matrix proteins associated with the fibrotic process [23]. Moreover, much evidence has shown that oxidative stress plays a critical role in HSC activation and hepatic collagen deposition (Figure 6) [24]. Recently, the relationship between liver fibrosis and oxidative stress level has attracted much attention. The oxidative stress produces excessive reactive oxygen species (ROS), which can react with membrane unsaturated fatty acids and cause lipid peroxidation leading to the formation of liver fibrosis [25,26]. In this study, it can be observed that all identified fatty acid markers are unsaturated fatty acids, including a monounsaturated (*i.e.*, oleic acid) and three polyunsaturated fatty acids (*i.e.*, docosahexaenoic acid, arachidonic acid and linoleic acid). Unsaturated fatty acids, especially polyunsaturated fatty acids, are more vulnerable to the free radical oxidation than any other biomolecules in lipid peroxidation [27]. Therefore, our finding of significantly decreased levels of unsaturated fatty acids in fibrotic rats indicates that the oxidative stress and resulting lipid peroxidation are involved in hepatic fibrogenesis.

In addition, various alterations of Lyso-PC levels were observed after DMN treatment. Lyso-PCs are the major product of phospholipid metabolism, with well-known membrane-toxic and proinflammatory properties [28]. In the metabolic cycle, various enzyme phospholipase types can catalyze the hydrolysis of phospholipids to release fatty acids and Lyso-PCs; meanwhile, Lyso-PCs can also be converted to phospholipids in the presence of lysophospholipid acyltransferase [29]. Therefore, the regulation of phospholipid homeostasis is crucial for the maintenances of normal liver function [28]. Because the liver plays a key role in blood Lyso-PC clearance and conversion, the altering Lyso-PC levels in fibrotic rats suggest an underlying phospholipid disturbance may be present in liver fibrosis.

### 2.4. Anti-Fibrotic Effects of YCHT

The present study demonstrated that YCHT intervention could ameliorate the DMN-induced liver fibrosis in rats. As seen in Figure 5, the levels of fibrosis-related markers, including all unsaturated fatty acids were significantly reversed by YCHT treatment (except Lyso-PC C20:4). These results implicate that YCHT exerts anti-fibrotic effects by regulating the disturbed lipid metabolism, especially through the suppression of oxidative stress and resulting lipid peroxidation in liver fibrosis (Figure 6). It has been reported that the caffeoylquinic acids in *Artemisia capillaries*, the iridoid glycosides and diterpenoid pigments in *Fructus gardenia*, and the tannins and polysaccharides in *Radix et Rhizoma Rhei* are the major active fractions of YCHT which synergistically contribute to its therapeutic effects against liver injury [30].

The main compositions of caffeoylquinic acids contain chlorogenic acid and its isomers or dimers [31]. All these components have various bioactivities such as antioxidant and anti-inflammatory effects. For example, the caffeoylquinic acids act as antioxidants can reduce lipid peroxidation by inhibiting the excess production of ROS in HSCs [32]. Therefore, the anti-fibrotic effects of caffeoylquinic acids are thought to be attributed to their beneficial roles in attenuating HSC activation. Meanwhile, inflammation is also considered an important event in fibrosis development [33]. Several studies have reported that chlorogenic acid could suppress the expression of proinflammatory cytokines (such as TNF-α and IL-6) in HSCs, which may also be due to the scavenging of ROS [34]. This suggests that the anti-inflammatory action of chlorogenic acid is one of the possible ways to improve liver fibrosis.

Geniposide is a major iridoid glycoside extracted from *Fructus gardenia* [35]. Previous results have indicated that geniposide can attenuate oxidative stress and resulting lipid peroxidation in liver [36]. Such antioxidant properties may be related to its ability to reduce free radical formation or its free radical scavenging activity. Moreover, genipin, an intestinal metabolite of geniposide after oral administration of YCHT, has been reported to work as an inhibitor of HSC activation [37]. This provides further evidence that YCHT may be useful for treating liver fibrosis by the absorbed form of its component. Besides, the main components of diterpenoid pigments in *Fructus gardenia*, such as crocin, have recently been shown to have protective effects on liver injury [38]. The mechanism for the efficacy of crocin is its ability to scavenge free radicals that causes oxidative damage to DNA, lipids and proteins.

Tannins are the major group of polyphenols contained in *Radix et Rhizoma Rhei*. The marker components of tannins, gallic acid and its derivatives, are shown to exhibit anti-fibrotic activity on liver [39]. As antioxidants and free radical scavengers, their mechanism of action might be through the inhibition of lipid peroxidation which plays a significant role in HSC activation [40]. These findings indicate that gallic acid may represent a potential therapeutic agent for liver fibrosis [41]. In addition, polysaccharide extracts are another class of active fractions isolated from *Radix et Rhizoma Rhei*. Recent evidence suggests that polysaccharides may protect the liver against fibrosis via its free radical scavenging and anti-oxidative properties [42]. Although the mechanism is still unclear, more efforts should be made to further understand their anti-fibrotic effects.

## 3. Experimental Section

### 3.1. Materials and Chemicals

The medicinal herbs of *Artemisia capillaries*, *Fructus gardenia* and *Radix et Rhizoma Rhei* were purchased from Shanghai Huayu Pharmaceutical Company (Shanghai, China). These materials were authenticated by a pharmacognosist, Dr. Fenghua Li, from Shanghai University of Traditional Chinese Medicine. The dimethylnitrosamine (DMN, 1 g/mL) was purchased from Sigma-Aldrich (St. Louis, MO, USA). The assay kits of alanine aminotransferase (ALT), aspartate aminotransferase (AST), Albumin (Alb), γ-glutamyltranspeptidase (GGT), alkaline phosphatase (ALP) and total bilirubin (TBil) were purchased from Nanjing Jiancheng Bioengineering Institute (Nanjing, China). 1-Palmitoyl-2-hydroxy-*sn*-glycero-3-phosphocholine (Lyso-PC C18:1) were purchased from Avanti Polar Lipids (Alabaster, AL, USA). Docosahexaenoic acid (C22:6), arachidonic acid (C20:4), linoleic acid (C18:2) and oleic acid (C18:1) were purchased from Sigma-Aldrichx. HPLC-grade acetonitrile and methanol were purchased from J. T. Baker (Phillipsburg, NJ, USA). Ultrapure water (18.2 MΩ) was purified with a Milli-Q system (Millipore, MA, USA). All other chemicals used were of analytical grade.

### 3.2. YCHT Preparation

YCHT was prepared as described in our previous study [11]. 18 g of *Artemisia capillaries* was soaked in 10 times its weight of water for 1 h, followed by decocting for 40 min. Then 10 g of *Fructus gardenia* and 6 g of *Radix et Rhizoma Rhei* were added and kept boiling for 15 min. The mixture was filtered and the filtrate was collected. The residue was then mixed with seven times its weight of water and further decocted for 20 min. After filtering, the two filtrates were combined and freeze-dried to powder at −50 °C (final yield of 28.8%).

### 3.3. Animal Experiment

All experimental procedures were approved by the Ethics Committee of the Laboratory Animal Center of Shanghai University of Traditional Chinese Medicine (project identification code: 20130704-4, approved: 4 July 2013). A total of 45 male Wistar rats (180 ± 10 g) were purchased from Shanghai SLAC Laboratory Animal Co. Ltd. (Shanghai, China). All animals were maintained in an air-conditioned room at 20–25 °C, relative humidity of 50%–70%, and 12 h dark/light cycle. The rats were fed certified standard chow and tap water *ad libitum*. After one week of acclimation, rats were randomly divided into three groups as follows (*n* = 15 in each group): the control, model and YCHT groups. The model rats as well as YCHT treated rats were injected intraperitoneally with DMN for four weeks, daily for consecutive three days each week at a dose of 10 mg/kg body weight to induce liver fibrosis. The control rats received an equal amount of physiological saline during the whole studies. At the beginning of the third week, the YCHT group was daily given YCHT orally at a dose of 0.418 g/100 g body weight, which is equivalent to the clinical human doses [11]. At the end of the fourth week, all animals were sacrificed and the sera and livers were taken for the following investigations.

### 3.4. Biochemical and Histological Tests

Sera of each group were collected from blood by centrifugation at 3000 rpm at 4 °C and stored at −80 °C for liver function tests. Serum levels of ALT, AST, Alb, GGT, ALP and TBil were tested on a Beckman Coulter Synchron LX20 biochemistry analyzer (Beckman Coulter, Fullerton, CA, USA). Liver hydroxyproline content were measured by the previous method [43], and the results were expressed as μg/g wet tissue.

The liver specimens were preserved in 10% neutral formalin and dehydrated in a graded alcohol series. The specimens were then embedded in paraffin blocks, cut into 5 μm thick sections and placed on glass slides. The sections were subsequently stained with Sirius red for light microscopic imaging.

### 3.5. UPLC-TOF-MS Analysis

The serum samples were prepared according to our previous works with minor modifications [21]. An amount of 100 μL of serum was used with addition of 400 μL of methanol. The mixture was vortex-mixed for 2 min followed by centrifugation at 6000 rpm for 15 min at 4 °C. The clear supernatant was transferred and diluted with ultrapure water (1:3, *v*/*v*). The solutions were filtered through a 0.22 μm syringe filter (Whatman, Florham Park, NJ, USA) before analysis.

A Waters ACQUITY UPLC system coupled with a Micromass LCT Premier™ orthogonal acceleration time-of-flight mass spectrometer equipped with an electrospray interface (Waters Corp., Milford, MA, USA) was used. Chromatographic separation was performed on a Waters ACQUITY BEH C18 column (100 × 2.1 mm, 1.7 μm) maintained at 50 °C. The mobile phase consisted of (A) 0.1% formic acid in water and (B) acetonitrile. A linear gradient was optimized as follows (flow rate, 0.4 mL/min): 0–3 min, 5% B to 50% B; 3–10 min, 50% B to 95% B; 10–11 min, 95% B to 5% B; 11–15 min, equilibration with 5% B. MS detection was operated in “W” optics (high-resolution) mode and the optimized conditions were as follows: negative ion (ESI−) mode, capillary voltage 2500 V, sample cone voltage 50 V, desolvation temperature 350 °C, source temperature 120 °C, cone gas flow 40 L/h, desolvation gas flow 700 L/h, and MCP detector voltage 2200 V. The data acquisition rate was set to 0.1 s, with a 0.05 s interscan delay using dynamic range enhancement (DRE). All analyses were acquired using the lock spray to ensure mass accuracy. Leucine-enkephalin was used as the reference lock mass (*m/z* 554.2615) at a concentration of 50 pg/mL and an infusion rate of 5 μL/min. Data were collected in centroid mode from 50 to 1000 *m/z*.

### 3.6. Analytical Method Validation

To ensure the reproducibility of the UPLC-TOF-MS method, both intraday and interday repeatability were validated based on the analysis of quality control (QC) samples. Aliquots of all sera were equally mixed together to serve as a pooled QC sample, which was then pretreated as described in Section 3.5. Intraday repeatability was assessed by analyzing six replicates of the QC samples at different times within one day. For interday repeatability, nine replicates of samples were measured thrice per day over three consecutive days. Relative standard deviations (RSD %) were calculated to evaluate the method reproducibility. Furthermore, ten QC samples were evenly inserted into the batch sequence to monitor the analytical performance.

### 3.7. Data Analysis

The UPLC-TOF-MS data of serum samples were firstly processed using MarkerLynx applications manager version 4.1 (Waters, Manchester, UK). After peak finding, alignment and filtering of the raw data, a list of the ion intensities of all compounds with their retention times and *m/z* values as identifier was generated. Ion intensities of each peak were normalized, within each sample, to the sum of the peak intensities in that sample. The resulting 3D data set were then exported to the software SIMCA-P+ 12.0 (Umetrics, Umea, Sweden). The data were pretreated using mean-centering and pareto-scaling prior to the multivariate analysis. PLS-DA analysis was applied to visualize the clustering among groups and identify the differentially expressed metabolites responsible for the separation. Statistical test was performed using the SPSS software (version 22.0, SPSS Inc., Chicago, NJ, USA). Groups were compared using one-way ANOVA analysis with Dunnett’s multiple comparison test or Student-Newman–Keuls test (where applicable).

## 4. Conclusions

In this work, an UPLC-TOF-MS-based serum metabolomic study was performed to investigate DMN-induced liver fibrosis in rats and the intervention effects of YCHT. After multivariate statistical analysis, a clear separation of the model and control groups was achieved, and the YCHT group exhibited a tendency to restore to the controls. Seven significantly changed fibrosis-related metabolites were identified, and most of their levels were also found to be reversed by YCHT treatment. The therapeutic effects of YCHT may be attributed to the inhibition of oxidative stress and resulting lipid peroxidation in liver fibrosis. These findings will contribute to increased understanding of liver fibrosis and the anti-fibrotic mechanisms of YCHT.
